# Using information communication technology in models of integrated community-based primary health care: learning from the iCOACH case studies

**DOI:** 10.1186/s13012-018-0780-3

**Published:** 2018-06-26

**Authors:** Carolyn Steele Gray, Jan Barnsley, Dominique Gagnon, Louise Belzile, Tim Kenealy, James Shaw, Nicolette Sheridan, Paul Wankah Nji, Walter P. Wodchis

**Affiliations:** 1 Bridgepoint Collaboratory for Research and Innovation, Lunenfeld-Tanenbaum Research Institute, Sinai Health System, 1 Bridgepoint Drive, Toronto, M4M 2B5 Canada; 2 Institute of Health Policy, Management and Evaluation, University of Toronto, 155 College St., Toronto, Ontario M5T 3M6 Canada; 3 Unité d’enseignement et de recherche en sciences du développement humain et social, Université du Québec en Abitibi-Témiscamingue, Val-d’Or, Canada; 4 Gerontology, Université de Sherbrooke, Sherbrooke, Canada; 5 South Auckland Clinical School, University of Auckland, Auckland, New Zealand; 6 Institute for Health System Solutions and Virtual Care, Women’s College Research Institute, Women’s College Hospital, Toronto, Canada; 7 Centre for Nursing and Health Research, School of Nursing, College of Health Te Kura Hauora Tengata, Massey University, Wellington, New Zealand; 8 Sciences de la Santé, Centre de Recherche—Hôpital Charles LeMoyne, Université de Sherbrooke—Campus Longueuil, Longueuil, Canada; 9 Implementation and Evaluation Science, Institute for Better Health, Trillium Health Partners, Mississauga, Canada

**Keywords:** Health information technology, Integrated health care systems, Implementation, Disruptive innovation, Chronic illnesses, Multi-morbidity

## Abstract

**Background:**

Information communication technology (ICT) is a critical enabler of integrated models of community-based primary health care; however, little is known about how *existing* technologies have been used to support *new* models of integrated care. To address this gap, we draw on data from an international study of integrated models, exploring how ICT is used to support activities of integrated care and the organizational and environmental barriers and enablers to its adoption.

**Methods:**

We take an embedded comparative multiple-case study approach using data from a study of implementation of nine models of integrated community-based primary health care, the *Implementing Integrated Care for Older Adults with Complex Health Needs* (iCOACH) study. Six cases from Canada, three each in Ontario and Quebec, and three in New Zealand, were studied. As part of the case studies, interviews were conducted with managers and front-line health care providers from February 2015 to March 2017. A qualitative descriptive approach was used to code data from 137 interviews and generate word tables to guide analysis.

**Results:**

Despite different models and contexts, we found strikingly similar accounts of the types of activities supported through ICT systems in each of the cases. ICT systems were used most frequently to support activities like care coordination by inter-professional teams through information sharing. However, providers were limited in their ability to efficiently share patient data due to data access issues across organizational and professional boundaries and due to system functionality limitations, such as a lack of interoperability.

**Conclusions:**

Even in innovative models of care, managers and providers in our cases mainly use technology to enable traditional ways of working. Technology limitations prevent more innovative uses of technology that could support disruption necessary to improve care delivery. We argue the barriers to more innovative use of technology are linked to three factors: (1) information access barriers, (2) limited functionality of available technology, and (3) organizational and provider inertia.

## Background

Older adults with complex care needs are among the highest users in health care systems worldwide [1–4]. This high use is not just about their complex health and social care needs which are more intensive as compared to other patient populations, but it is also due to health care systems lacking the necessary structure to effectively and efficiently support this population [5]. Although no one definition of *complex care needs* is available in the literature, we define this group as individuals with multiple chronic conditions (e.g., multi-morbidity), commonly accompanied by socio-economic deprivation, and a largely unpredictable, changing evolution of care need, that makes the management of their illness particularly challenging [6–10].

Integrated care delivery, which brings together and coordinates services from across the health and social system, has been identified as critical to meet the needs of this patient population [11], preventing unnecessary use of health and social care resources. Information and communication technology (ICT) has been identified as an important enabler to support delivery of integrated and coordinated primary health care [12–17]. ICT enabled information sharing across professional and organizational boundaries is arguably one of the most crucial aspects of successful models of integrated care [13, 15].

While suggestions describing optimal ICT systems are common in the literature, to our knowledge, there are few studies that have explored how ICT is used to enable the activities necessary for integrated care. We do not have a clear understanding of how ICT has been adopted in practice in real-world implementations of integrated community-based primary health care and how organizational environments play a role in that implementation. To address this gap, this study draws on qualitative data from an international study of models integrated care to answer two research questions:

1. What functionality, use, and role does ICT play to enable activities of integrated models of community-based primary health care?
2. What are the implementation enablers and challenges in adopting ICT across different organizational contexts?

To address the first question, we need to clearly identify integrated care activities after which we can determine the role of ICT systems in enabling the implementation of those activities. Wagner’s *Chronic Care Model* has been used as a guide to deliver care to individuals with chronic and complex disease in primary care settings [18]. The model includes elements that are well aligned to core activities and components of integrated care including self-management, multi-disciplinary teamwork, and decision supports [19]. Over the years, the Chronic Care Model (CCM) has been revised, expanded, and enhanced in multiple ways to address identified gaps or apply the model to new contexts (Wager’s expanded model in 2003 for example [20]). A useful augmentation to the model for our purposes is the *eHealth Enhanced CCM* (hereafter referred to as the eCCM), a framework identifying how ICT tools can be incorporated into the CCM and used by providers and patients in the delivery of care [21]. The eCCM is built on a core assumption that sharing information will generate knowledge and collective wisdom among health care providers and managers which can be used to improve health outcomes for patients.

Several types of ICT tools used to exchange health information are identified in the eCCM and broader eHealth literature including (1) electronic medical and health records (EMRs/EHRs), (2) patient personal health records (PHRs), (3) telemonitoring systems (using phone, mobile, and sensor-based technology), and (4) web-based resources (e.g., educational sites and social networks) [22–27]. Table 1 summarizes Wagner’s CCM elements and connects them to ICT enhancements suggested in the eCCM.

**Table 1:** Summary of ICT-supported elements of the CCM

CCM element	Description	ICT-supported elements
Community resources and policies	Providers connecting patients to community programs through partnerships that expand health system services beyond primary care. Providers promote patient self-help strategies, including connecting to real and virtual social networks.	Health-related social networks and eCommunities that support health and social care connections.
Health system	Designing health systems to support organizations and providers in their interactions with patients around chronic disease care. Organizational and senior leadership support creating a culture of safety and improvement across all organizations that make up the health system. Including supporting data sharing across the system to improve chronic disease care.	EHRs, PHRs, mHealth and Telehealth, online resources/systems that support quality improvement and patient engagement.
Delivery system design	Adopting a proactive patient management approach through care coordination and case management, especially for complex patients. Includes provider team members having clear roles and responsibilities, ensuring regular follow-up, and care that patients understand, find acceptable and fits with their cultural background.	EHRs, PHRs, mHealth and Telehealth that enable information sharing and span the system.
Self-management support	Supporting patients with chronic illnesses to make decisions and engage in actions that improve their health (self-management). Providers collaborate with patients to define problems, set goals, and create treatment plans (self-management support strategies).	PHRs, online resources/systems, mHealth and Telehealth, and applications that support patient-provider interactions.
Decision supports	Supporting the use of evidence-based guidelines into daily practice which can be shared with patients. Education and training for providers and integration of specialist expertise included in this element. New models of provider education in particular eHealth Education (added in the eHealth enhanced CCM): encourages the development of eHealth skills for patients and providers that can enhance all six elements of CCM.	Electronic access to evidence-based guidelines, protocols, standing orders, and reminders for providers and patients, through EMRs, EHRs, or online resources. Appropriate training for providers and patients on eHealth Education systems.
Clinical information systems	Information systems providing ready access to key data on individual patients—reminders for services and data to track and plan care—and at the practice level, population data to monitor performance and improve quality, in particular for relevant subpopulations.	EMRs, EHRs, and PHRs that support coordinated care and monitoring performance at the individual level and practice level.

While the eCCM is a useful guide regarding how we might expect ICT to be used as part of integrated models for patients with complex care needs, it mainly offers a high-level view with few examples of specific provider and organizational level activities associated with these elements. Our study takes the next step by exploring how the *activities* associated with each of the CCM elements are, or are not, enhanced through the adoption of ICT systems.

Our second research question seeks to understand *why* ICT is or is not used to support these activities. There are a number of organizational change and implementation theories that have been applied to better understand the adoption of ICT and eHealth technologies. In our review of the literature, we identified five theories of implementation science which have been used to explore implementation of ICT in similar health care settings (Roger’s Diffusion of Innovation; Normalization Process Theory; the Reach, Effectiveness, Adoption, Implementation, and Maintenance framework; the Fit between Individual Task and Technology; and the Consolidated Framework for Implementation Research) [28–37]. While some other theories have been adopted, these five were most prominent in our search of the literature at the time the analysis was conducted for this study.

The five models and theories differ in terms of specific constructs and theoretical underpinnings. For instance, Diffusion of Innovation theory stems from the organizational behavior and change literatures, whereas Normalization Process Theory focuses on the social organization of work. Constructs included in these different frameworks offer different perspectives on a similar concept. Taking the individual characteristics constructs across theories for example, we see in the Consolidated Framework for Implementation Research an emphasis on *individual level* processes such as knowledge and beliefs and their degree of commitment to the organization [32], whereas Normalization Process Theory focuses on the *interaction between individuals and their social environment* or “material practices” that may become routine [29]. The Fit between Individual Task and Technology, on the other hand, acknowledges the role of the individual in relation to their work, but specifically with how a new model fits to existing practices [31]. Each theory suggests a different perspective of the individual in the implementation. Our previous work in concept mapping to inform the iCOACH study has shown that drawing on different theoretical perspectives will allow for a more in-depth and varied understanding of meanings and values assigned to seemingly similar constructs [38]. Combining theoretical frameworks ensures we are not missing these varied perspectives of participants in our analysis of findings.

Looking across the theoretical frameworks, we sought to consolidate constructs into core domains expected to influence ICT adoption in models of integrated care. To develop the domains, we looked across theories at how constructs are defined and operationalized in previous studies of ICT adoption in health care. A table was generated mapping constructs from the theories to the domains and was agreed upon by the co-authors. We have used similar methods of concept mapping when adopting multiple theoretical frameworks [38]. Using this method, we identified four core domains: (1) *characteristics of individuals* adopting technology, (2) the *organizational* and (3) *external environment*, and (4) the *characteristics of the technology*. Table 2 presents a summary of these domains and examples of factors which are pulled from across the five different theoretical frameworks. Using the four core domains, we are able to explore which implementation factors have an essential or peripheral role in the adoption of ICT systems in real-world environments where models of care are often implemented without an explicit ICT strategy in place to support the core activities and aims.

**Table 2:** Domains of implementation of information communication technology in health care settings

Domain	Definition and origin	Examples of factors/determinants
Characteristics of individuals	Individual-level knowledge, beliefs, self-efficacy, and cognitive process that influence understanding, trust, and adoption of technology *NPT* (emphasis on social processes and cognition), *FITT*, *CFIR*, *DOI*, and *RE-AIM*	Knowledge, beliefs, attitudes, and norms Self-efficacy eHealth literacy/training Personal traits (motivation, values) Participation/engagement Task/work coherence Adherence
Organizational environment	Characteristics of the organization such as organizational-level culture and availability of resources that influence ICT adoption. *DOI*, *CFIR*, *FITT*, *NPT*	Technical support Organizational size Organizational culture and climate Readiness for change Routinization of use Organization of tasks and activities and complexity of task
External environment	Macro-level features surrounding organizations and networks including political, economic, and social contexts. *DOI*, *CFIR*	Regulations and policies around ICT use Funding Organizational interdependence Location (urban vs. rural setting) Patient and population health needs
Characteristics of technology	Attributes of the technology which will (or will not) fit with the needs of users and the attributes of the organization and environment. *FITT*, *DOI*, *RE-AIM*, *CFIR*, *NPT* (characteristics of intervention more broadly)	Usability (effectiveness, efficiency, learnability, satisfaction) Functionality (including adaptability of features) Cost Integration Available technical infrastructure Availability

*NPT* Normalization Process Theory; *FITT* Fit between Individual, Task and Technology; *CFIR* Consolidated Framework for Implementation Research; *DOI* Diffusion of Innovation; *RE-AIM* Reach, Effectiveness, Adoption, Implementation, and Maintenance

## Methods

### Approach and design

Our study takes an embedded comparative multiple-case study approach [39, 40] using data collected as part of the *Implementing Integrated Care for Older Adults with Complex Health Needs* (iCOACH) study. The iCOACH project is a multi-year international study exploring the implementation of nine models of integrated community-based primary health care across three jurisdictions, Ontario and Quebec in Canada and New Zealand. Background on this study is available in a special issue of the International Journal of Integrated Care published in June 2017 [41]. Cases that had implemented integrated community-based primary health care were selected for the broader iCOACH study [42]. While there are some key examples where technology has been developed alongside the model of care [43], the iCOACH study did not select cases based on this strategy. As such, these cases provide examples of how *existing* technologies have been used to enable *the implementation* of models of integrated care, offering a unique opportunity to explore our research questions. While the broader case study includes qualitative as well as quantitative data sources, for the study presented in this paper, we draw on qualitative interviews with managers and front-line health care providers collected between February 2015 and March 2017 with the majority of interviews conducted in 2015 (Quebec case data collection ran a bit later than Ontario and New Zealand).

To answer our first research question, we use an embedded cross-case analysis, looking across the different models of integrated care within each jurisdiction to identify and describe activities of providers and managers that are (or are not) enabled by the use of ICT. We then conduct a cross-jurisdictional exploratory analysis, allowing us to take into consideration both organizational and external environments in the investigation of implementation enablers and barriers addressing our second research question.

### Setting: the nine cases

In-depth descriptions of all nine cases and three jurisdictional policy environments are available through other iCOACH publications [44, 45]. This section offers a brief summary of key contextual factors relevant to our analysis with additional data presented in Table 3. Consistent across all three cases are strong legislative and regulatory policies protecting personal health information privacy and security, as well as a general interest by regional and national governments to adopt technology to support health system delivery.

**Table 3:** iCOACH case characteristics

	Ontario			Quebec			New Zealand		
Cases	Community agency lead (case 1)	Primary care and home care (case 2)	Community health center (case 3)	Highly urban (case 1)	Urban (case 2)	Rural (case 3)	Network model (case 1)	Māori (indigenous) NGO (case 2)	PHO home visiting program (case 3)
Model	Single organization Multiple services available including primary care services Partnerships with hospitals and home care delivery around contracts and/or projects	Partnership model Multi-disciplinary primary care practice and home care delivery agency Strong connections to local hospital, emergency services, and community agencies	Single organization Multiple services under one roof including primary care Community-focused with an emphasis on social determinants of health Co-location through hubs with community service partners	Regional model with 1 hospital, 3 long-term care facilities, and 2 local community health centers (CLSC) Connects to primary health care, community agencies, rehab, pharmacies, and private residences	Regional model with 4 long-term care facilities and 4 local community health centers (CLSC) Connects to university teaching hospital, primary health care, community agencies, rehab, pharmacies, and private residences	Regional model with 1 hospital, 1 long-term care facility, and 1 local community health center (CLSC) Connects to primary health care, community agencies, rehab, pharmacies, and private residences	Network of urban and rural practices within a single district health board Programs create consensus care pathways with implementation across primary care and secondary care. Multi-disciplinary primary/community care providers Services to support integrated care for older adults with complex conditions (e.g., home services to enable early hospital discharge)	Community-owned NGO providing public health and primary care services to urban and semi-rural populations experiencing high material deprivation. Inter-professional team (doctor, nurse practitioner, nurse, pharmacist, navigator) Whanau (family) navigator works with patients and families to access health and social services	Chronic care management program for patients in 1 of 6 rural or semi-rural primary care practices Teams of a nurse and a kaiawhina (community health worker) home visit patients to provide clinical assessment, education, and coordinate health and social services Service typically offered for 6 months
Provider interviews	8	8	7	14	8	7	4	7	8
Manager interviews	8	10	6	12	9	11	6	2	2

Ontario community-based primary health care includes services from both health and social care sectors [45]. Services in Ontario are often siloed with few integrating mechanisms available. Fragmentation in the delivery system extends to health information systems [46], which is exacerbated by Ontario’s low rules policy environment (allowing multiple vendors to compete for contracts across health care organizations), and tight information privacy and security legislation [47]. For the most part, data systems exist within single organizations in practice and organizational-based EMRs. One notable exception is the CRIS system which captures data for patients receiving provincially funded home care services. None of the Ontario cases include an explicit ICT strategy or system in the initial implementation of the model, but rather use and adapt existing systems.

In Quebec, the health and social services sectors have been under the same governance, with a centralized government-initiated implementation of integrated care models since 2004. As part of this strategy, Quebec has in place two central ICT systems: (1) RSIPA (available to home support professionals as well as to various organizations within the same local services network) and (2) I-CLSC Customer and Service Information System (containing health administrative data). Despite these two centralized IT systems, like Ontario, there is no single clinical record in Quebec, but rather separate EMR systems connected to health care clinics. Several databases developed by the private or the public sectors have been purchased by health and social services institutions.

Similar to Ontario and Quebec, service delivery and funding in New Zealand are separated by region. Distinct to New Zealand is the unique identification code assigned to each person that can be used to link individuals’ health information across any hardware and software platforms. New Zealand has long had a high rate of computer use in primary care [48] with essentially 100% of primary care using an EMR. ICT use in primary care has typically been ahead of use in secondary care, with a proliferation of add-ons to the primary care systems, including well-integrated browsers running other programs in real time. The three cases in New Zealand are similar to those in Ontario in that they represent more local solutions to community needs for integration, rather than a provincial strategy as in Quebec.

### Data collection

Interviews with managers and front-line health care providers lasted between 30 and 90 minutes (most lasting about 60 minutes) and were all transcribed verbatim. Interviews from Quebec cases were conducted, transcribed, and analyzed in French by French-speaking members of the research team. During the interviews, providers were asked specifically about their use of ICT systems as part of the delivery of care, and managers were asked about key implementation factors of their integrated care models in their organizations or networks. As part of the manager interviews, participants were provided with a framework of implementation factors likely to impact on the adoption of their models of care (the Organizational Context and Capabilities for Integrating Care framework [49]), which includes ICT as a factor. Data from 137 interviews with managers (*n* = 66) and providers (*n* = 71) is included in our analysis. Table 3 summarizes the number of interviews across each case. Providers in each case could include physicians, nurse practitioners, registered nurses, social workers, dietitians, pharmacists, physician assistants, and personal support workers. Managers in each case could include executive directors of clinics, clinical leads, directors and managers in human resources, strategic planning, and programs. Ethics approval for this study was provided by all necessary ethics boards within each jurisdiction.

### Data analysis

To conduct our embedded and cross-jurisdiction analyses, we use a cross-case synthesis analysis approach using *word tables*, a method used to “display data from individual cases according to some uniform framework” [40] (p. 156); in this case, our uniform framework is derived from keywords and concepts from the eCCM and the implementation frameworks presented in Tables 1 and 2. To generate word tables for this study, we first extracted data from interviews with organizational managers and providers from each of the nine case sites. Qualitative descriptive [50] coding was done across all transcripts as part of the broader iCOACH study analysis strategy which included an IT/ICT code. Node reports from this theme were extracted and coded first using thematic content analysis to map content [51]. Coding was iterative and repeated by multiple members of the research team to support dependability and credibility [52] of the coding structure and code descriptions. Coded data was then applied to the eCCM and implementation frameworks to populate word tables.

Given the volume of data, study results are presented on aggregate at the case or jurisdictional level, with some exemplary quotes provided. Study results are presented in two sections: The first focuses on the embedded case-analysis related to our first research question; the second presents the cross-case comparative analysis at the jurisdictional level, addressing findings related to our second research question.

## Results

### Functionality, use, and role of ICT: embedded-case level analysis

The embedded-case level analysis reveals a number of activities associated with elements of the CCM model presented in Table 1. Despite different models and contexts in care, we found strikingly similar accounts of the types of activities that were supported through ICT systems in each of the cases. Table 4 summarizes the ICT-enabled activities aligned with each of the elements of CCM as seen in our cases. We follow this with a more nuanced examination of the activities within each element as they manifest in each of the cases. Our findings represent accounts of providers and managers of what and how ICT systems *are used* to support their particular models of care, rather than presenting an account of all *available* ICT systems in each jurisdiction.

**Table 4:** Summary of CCM element activities supported by ICT identified in nine cases

CCM element	Activity	ON case 1	ON case 2	ON case 3	QC cases 1–3	NZ case 1	NZ case 2	NZ case 3
Community resources and policies	Information sharing (health to social care)		•		•	•	•	•
Health system	Informed management decisions				•	•	•	
	Quality improvement	•	•	•		•	•	
	Case finding	•	•			•	•	•
	Action planning				•	•	•	•
Delivery system design	Care coordination	•	•	•	•	•	•	•
	Assessment				•	•	•	
	Electronic referral	•				•	•	
	Remote monitoring		•			•	•	•
	Prevention activities	•			•	•	•	•
Self-management support	Part of care planning				•	•	•	•
	Collaborative decision-making		•		•			•
	Provide access to resources					•	•	•
Decision supports	Clinical guidelines				•	•	•	
	Referral pathways					•	•	
	Peer support					•	•	
Clinical information systems	Documentation	•	•	•	•	•	•	•
	Clinical level	•	•	•	•	•	•	•
	Regional level				•	•	•	•

Activities supported by ICT as identified by participants. This may not be a complete list of all activities conducted by cases, or across their respective jurisdictions, in relation to these elements of the CCM. The table is intended to summarize the findings discussed in the following section

ON case 1—community agency lead; ON case 2—primary care and home care partnership; ON case 3—community health center; NZ case 1—network model; NZ case 2—Maori NGO; NZ case 3—PHO home visiting program

#### Community resources and policies

The community resource and policy element suggests systems support connecting patients to community programs through the development of partnerships between health and social care services [18, 20]. The eCCM suggests this is mainly supported through eCommunities; however, our cases used ICT a little differently to enable this connection. Quebec, New Zealand, and one case in Ontario used ICT to support information sharing and follow-up between sectors, most often enabled through giving community providers access to primary care EMR systems. For the most part, Ontario and New Zealand cases connected primary care to community resources through means other than ICT, drawing mainly on embedded professionals (like the Whanau Ora workers in New Zealand case 2 who linked health and social services), and personal relationships and informal connections between providers.

#### The health system

The health system element suggests health systems be organized to enable the delivery of chronic disease care to patients [18, 20]. Cases in our study used ICT systems to support informed management decisions (helping to plan and fund care delivery in a region), quality improvement initiatives (at both organizational and clinic levels), and case finding (identifying population groups to target with initiatives, e.g., chronic disease lists in EMR systems) as a means to improve chronic disease care. A notable difference between jurisdictions is that these activities were enabled primarily using clinic- and organizational-level patient data in Ontario, whereas regional-level data were available in Quebec and local, regional, and national data were available in New Zealand (e.g., RSIPA in Quebec and district and national data in New Zealand).

#### Delivery system design

The delivery system design element of the CCM refers to adopting proactive patient care through activities like case management and care coordination by inter-professional teams [18, 20]. ICT systems in our cases were used most frequently to support the delivery system design element, in particular, care coordination activities supported across all nine cases. In Ontario, ICT was used to share patient information within teams who could access clinic-level EMR systems. Information sharing was both asynchronous (e.g., looking at another provider’s notes about a patient) and synchronous (e.g., using a messaging system or calling another provider to discuss patient care). Quebec and New Zealand had much more robust systems to support care coordination as patient data were stored on both clinic and regional level systems and included patient assessments and care plans to support coordination and prevention activities. ICT was also used to support remote monitoring, eReferrals, and virtual consults in some cases.

#### Self-management support

The self-management support element emphasizes the patient’s role in their health management and refers to activities like assessment, care planning, and follow-up [17, 19]. In the iCOACH cases, each of these activities was supported by ICT systems, often in relation to activities of care coordination and case management reflected in the previous section. For instance, in the New Zealand cases, patients and providers typically collaborate in the care planning process which is then recorded into the EMR. However, other forms of self-management support, notably collaborative goal setting, were not often discussed in relation to the use of ICT within the case interviews (although goal setting may be used in the care planning process, this was not explicitly within ICT-related data). ICT systems were more likely to be used to help provide patients with information about their illness.

#### Decision supports

Decision supports refers to promoting the use of evidence-based guidelines in the delivery of care [17, 19]. Regional- and practice-level EMRs in Quebec and New Zealand supported the use of evidence-based guidelines. In New Zealand, clinical guidelines, referral pathways, and operation manuals were all available on practice EMR systems. However, the degree to which these systems are embedded into the EMRs varies between guidelines and practices ranging from auto-calculation of cardiovascular risk and prompting for therapy option (fully embedded) to interactive or passive access pathways to access via the web to collections of high-quality guidelines from anywhere in the world. In Quebec, standardized clinical evaluation tools can be accessed remotely for nurses conducting home visits in the highly urban area. In Ontario, physicians in two of three cases mentioned using an online system, called Up-to-Date, run by their provincial professional association to support the use of evidence-based guidelines as a decision support tool.

#### Clinical information systems

Clinical information systems in the CCM framework refer to the use of information systems to organize patient and population health data [17, 19]. Important to note are the ICTs in Ontario and New Zealand cases are constructed around practice-level EMR systems, as compared to Quebec which had more available regionalized systems to document patient encounters and inform clinical care.

### Implementation challenges and opportunities: jurisdictional-level analysis

The analysis of implementation factors helps us understand why we see a convergence around some ICT-enabled activities over others. Implementation challenges across the three diverse jurisdictions were also remarkably similar with regard to the type of implementation constructs, and are summarized in Table 5.

**Table 5:** Summary of implementation factors identified in iCOACH study, by jurisdictions

Implementation constructs	Ontario	Quebec	New Zealand
Characteristics of individuals	Personal values/norms Task/work coherence eHealth literacy	Personal values/norms Task/work coherence eHealth literacy Training	Personal values/norms Task/work coherence eHealth literacy Training
Organizational environment	Organizational policy and culture Technical support	Organizational culture and climate	Organizational policy and culture Technical support
External environment	Regulations and policy (privacy, data access) Connections between organizations	Regulations and policy (privacy, data access) Geographic location (rural setting)	Regulations and policy (privacy, data access) Cost/funding
Characteristics of technology	Functionality (interoperability)	Functionality (interoperability) Usability	Functionality (interoperability) Usability

Closer examination of each construct reveals important variation which can be linked to jurisdictional and case level environments. Notably, implementation constructs often interrelated and overlapped in important ways. Rather than interrogating each construct presented in Table 5, this section walks through one *implementation narrative* as expressed across three different jurisdictions. We focus specifically on how the task of *information sharing* to support care coordination (identified in the previous section as one of the activities best supported by ICT) was enabled and hindered by individual, organization, environmental, and technological level implementation factors.

#### Ontario—information sharing in a fragmented system environment

ICT systems enabled information sharing to support care coordination when needed data could be accessed quickly and easily by the providers during their usual workflows. In Ontario, providers and managers reflected that having patient data in a centralized place (a single EMR system) accessible by all team members was critical to deliver coordinated care:

You don’t have to go looking for these paper charts all over the place. You have a centralized place. You go in there, you find everything that you need about the client. (Nurse practitioner, ON, case 3)

Unfortunately, patient data was, for the most part, barricaded within the walls of each organization, with little information flowing between different organizations. A notable exception was the Hospital Report Manager system which created a connection from hospital to primary care EMR practices. While a step in the right direction, these systems did not allow for *two-way* communication, and not all hospitals were connected to all practices so data could be missed.

The great thing is our connection to [local hospital] which is only about a year old now. Having that direct integration of reports is amazing […] Like, if we could speak with other hospitals the way we do with [local hospital], and in my dream work, if we could speak with pharmacies at all. (Pharmacist, ON, case 2)

The limitations in connectivity and interoperability between systems resulted in providers and managers engaging in work-arounds to retrieve information needed to coordinate care. In Ontario, providers from different settings would co-locate so they could access multiple platforms through different portals. Mostly, providers continued to use phone, fax, and face-to-face interactions to share information. While these interactions were important to team relationship building, they also created inefficiencies and increased likelihood of missed information.

#### Quebec—information sharing in a centralized model

Of all cases, Quebec represents the closest example of strategic and purposive ICT adoption intended to support integrated care. Specifically, the RSIPA system used in home care was put in place explicitly to enable care integration by supporting information sharing. Beyond RSIPA, clinical information could also be housed and shared through organization-centric systems like EMRs or SYMO (an information system used by nurses). As was the case in Ontario, access to this information was highly valued by providers as expressed by one provider speaking about SYMO:

If I visit the patient of a colleague.... no matter what kind of care, then I realize that something not normal, then there, for a moment. I remove my gloves, I check the computer, I look, and then I can see all the previous notes of this procedure, which was done at home. Then there, ah, my sweet, yes, it’s known, the doctor has been warned, it’s beautiful. It’s correct. (Nurse, QC, case 1)

Even with regional systems in place, information sharing worked best when it occurred between providers working in the same organization within the same professional groups, as interoperability between ICT platforms remained elusive. Compounding this issue were professional level data access problems so that even providers within the same territory in the same organization could not see all shared patient information. For example, a social worker in a community health center did not have access to other providers’ patient progress notes, and hospital providers were unable to access nursing notes of homecare providers when parallel systems (like SYMO) are used. Data access problems hindered case managers’ ability to coordinate care.

So they [nurses] have access to my computerized patient notes, […] While I do not have access to the details of their [patient] notes, but ... but given my role as a case manager, it’s a big barrier. (Social worker, QC, case 2)

Like Ontario, providers engaged in different types of work-around strategies to meet their information sharing needs. Providers would often resort to written notes and faxing when the electronic systems were insufficient.

#### New Zealand—information sharing in a multi-system space

The New Zealand digital assets provided robust patient data available on multiple platforms. Providers are able to quickly find patient information as patients are assigned unique identifiers used across all different systems. Data access supported inter-professional teamwork; particularly where there were ICT-enabled care plans to help ensure continuity.

Sometimes the doctor might have sent a task message, to me, to let me know from something that’s going on. Or, I might have if there was something that I’ve put in the notes that I think’s more important that the doctor knows about, I’ll send them a message and often they might send a response back. (Provider 19, NZ, case 3)

While there was a lot of patient information available to providers, this data existed on multiple systems. So while a single system may be able to integrate important forms (like care plans) and reminders (supporting preventative care), a provider may need to access multiple systems to get a full picture of the patient. While not always perceived as problematic by providers, it does demonstrate important inefficiencies within the system:

And then, I take all that information from it and then I try to transfer that as clearly and as concisely as I can onto the two computer systems, copy and paste, onto CCMS, over to [regional information system], so that if a GP is… have time to sit there and think, ‘Oh I might have a look at that client,’ out of the blue, which is a reality. (Provider 08, NZ, case 1)

Similar to the challenge in Quebec, different professional groups did not always have access to data across specific professional platforms.

I’ve found I’m kind of locked out of in terms of being able to send electronic referrals off. It requires me to punch in a doctor’s registration code, which is a bit of an issue, in terms of the system that we use. We’re still able to work around that, it’s just a… one of a few different hassles. (Provider 04, NZ, case 2)

As in Ontario and Quebec, these interoperability and access issues resulted in providers engaging in work-arounds. Providers would print information and in some instances would learn coding and how to develop new forms in the systems to better meet their information needs. There were more instances of technological work-arounds identified in the New Zealand cases where the providers appeared to be better trained on systems and more tech-savvy as compared to the Quebec and Ontario case sites.

## Discussion

Study results suggest that ICT systems are primarily being used to support coordination of care through information sharing, resonating with the literature on the appropriate role of technology in these models [13, 15]. Findings also support the claim of the eCCM that information sharing is crucial to this model of care. However, the full potential of these systems to support other critical activities of integrated care are not being fully realized, which can be attributable to implementation determinants as depicted in the three narratives presented. We explore these determinants from the perspective of integrated care as examples of a disruptive innovation [53, 54] in the health system, as the model alters the status quo in health care service delivery with an aim of higher quality more efficient care delivery [55, 56]. Where eHealth is argued to be a *driver of innovation* [17], our study suggests that technology use in these cases remains limited to old ways of working, often creating process inefficiencies and the need for complex workaround strategies. In effect, providers are *following* old ways of working rather than *leading* through the adoption of more innovative uses ICT to support the new models of care. We put forward that the barriers to more innovative adoption of ICT are linked to (1) data access barriers, (2) limited functionality of available technology, and (3) organizational and provider inertia.

### Data access barriers

All three jurisdictions reported difficulties with accessing patient information, even when robust information systems were available. In the Ontario cases where there were no accessible regionalized datasets for the purposes of care delivery, providers continued to share information within organizational silos and rely on old ways of working for cross-organizational information sharing. Managers reflected that this limitation was a critical barrier to scale and spread of the model. In Quebec and New Zealand, we find differing access to data depending on profession and health sector, resulting in providers within the same models of care not being able to see all available information about shared patients. There are also additional administrative burdens to patients and providers in these jurisdictions, such as patients having to sign multiple consent forms and regular information use audits. Despite the extra steps, strict rules of data security were still highly valued by providers, building trust that their patient data was secure.

Thorough consent and audit systems used in our cases are often recommended to ensure data security for shared patient EHR systems [57]. All three jurisdictions have strong data privacy and security regulations; while critically important, stringent regulatory environments like this have been linked to slow diffusion of technology and a general hindrance to innovation [58]. Furthermore, these regulations may not reflect patient needs, as complex patients tend to be less concerned regarding privacy and security of their own data [59] and often just want all their providers to know their information [60]. There are good examples of robust health information exchange systems in the USA that can be looked to for guidance in terms of setting interoperability standards and patient consent processes [61]. The remedy for this barrier to innovation, we argue, is not in weakening security systems, but rather in re-considering unintended consequences and establishing processes that ensure data security without jeopardizing innovation.

### Limited technology functionality

In addition to limiting policy environments, integrated care activities in our cases are confined to available functionality of systems. Across all cases, a lack of interoperability between organizational and regionally based systems was one of the most important challenges; for Quebec cases, this is perceived as more important than the limiting policy environment. Lacking interoperability is concerning as integrated information systems are viewed as a prerequisite for integrated care [12–16]. In a study of 27 European models of integrated care, interoperable information systems were found to be necessary for sustained adoption [62]. Shifting to an interoperable system at an inter-organizational level is both a technological and linguistic challenge as it requires large-scale organizational change relating to setting information standards [63]. Another option would be to develop a centralized system (preferably with a single ICT vendor), which Quebec has done to some degree but not fully. One prominent example of a centralized data system is that used by Kaiser Permanente in the USA [64, 65].

Managers and providers also identified numerous usability challenges across cases. As noted in the findings, systems were inefficient and often did not fit user workflows. User-centered design approaches can address this challenge through incorporating users perspective into the design of new systems through an iterative approach [66, 67]; these methods have been found to improve user adoption, acceptance, and satisfaction with new systems and can also contribute to improved implementation overall [68, 69]. User-centered design works well when existing work processes are effective and require little change; however, when practices require more drastic alteration, other, more disruptive approaches to design and implementation may be required.

### Organizational and provider inertia

In the nine iCOACH cases, ICT systems were mainly used to support information sharing as a means to enable care coordination across teams and organizations. With the exception of a few more inventive uses of ICT, for example, the use of eConsults with specialists and virtual visits with homebound patients, providers generally used ICT as electronic patient information filing systems. Yet, other critical activities of integrated care such as prevention, collaborative goal setting, and community resource integration could be well served by innovative ICT solutions. Technologies like wearables [70], mobile systems [71], telemonitoring [72], and virtual teams [13] could all be beneficial to models of integrated care and are often seen as disruptive to usual care [73].

The organizational behavior literature sheds some light on why organizations and providers may be resistant to innovation. First, previous studies of organizational innovation adoption have found that even when organizations search for innovations to improve processes, what they tend to adopt supports “more traditional strategic and operating changes” [74] (p. 72). This suggests that organizations have a tendency towards using technologies to support practice as usual, which may account for the ICT adoption we see in the iCOACH cases.

Second, disruptive innovations are inherently risky. It has been found that organizations are more able to innovate when there are “safe spaces for actors to experiment with new ideas and develop new ways of working together” [75] (p. 212). Indeed risks can be significant as new ICT systems can lead to initial reductions in efficiency and impacts on worker autonomy [76]. Christensen suggests organizations hive off smaller offshoots to enable this type of more risky experimentation [53]; however, this strategy is not typically available for health care organizations.

One method used in other integrated models has been to explicitly develop enabling ICT systems alongside new models of care. The ICT system in Catalonia (Spain) for example was explicitly developed to support better integration of health and social services [77] and includes much more robust ICT solutions, along with an expectation that providers will also change behaviors [78]. With more explicit planning and development, ICT could be more effective as a tool for purposeful and positive disruption.

### Limitations and future directions

The cases presented in this study constitute real-world implementations of integrated community-based primary health care that used primarily existing ICT solutions. The systems used by the cases, however, do not represent *all* available ICT efforts within each jurisdiction, and as such, this analysis is not intended to suggest that the uses represent all available technologies that *could be* used to support the model of care. Similarly, the implementation factors discussed by participants may not fully capture implementation factors relevant to all implementations of integrated care. Our intention was not to be exhaustive, but rather to extract implementation factors most important to our cases as represented by the perspectives of our participants. Important to consider is the study presented in this paper is both cross-sectional and exploratory. What we offer are the activities and implementation factors discussed by participants, but additional work will need to be done to apply underlying theoretical foundations to build an explanatory model. In order to fully realize the potential of applying a multi-theoretical perspective of implementation factors, we recommend adopting a longitudinal and ethnographic approach, which can allow for an analysis of how implementation factors related to ICT use in integrated care models can change over time with particular attention to the how these factors *determine* use and adoption. This type of design could allow for a deeper dive into theoretical underpinnings driving processes like the provider and organizational change, for instance, by following providers’ adoption of ICT over time and observing their individual and social interactions around ICT. A longitudinal design could also serve to test and expand upon the framework.

A final limitation is that our theoretical framework was based on a literature review of implementation theories and models used to explore and study the adoption of ICT in health care settings, rather than a systematic review. We may have missed some implementation models that could expand our understanding. One notable model identified after the analysis was completed is the Theoretical Domains Framework, a psychological framework of behavior change [79]. An important next step would be to conduct a systematic review of theories to inform future work.

## Conclusions

Providers and managers working in integrated community-based primary health care models are adopting ICT to enable some key activities in the model of care but do so in a limited way which primarily supports existing ways of working. Extending from this work, as well as the experiences of the co-authors in this field, we offer three recommendations to decision-makers and leaders seeking to adopt ICT systems in models of integrated care: first, is to work with organizations and decision makers, such as regional privacy officers to mitigate policy and regulatory barriers; second, is to find a balance between user-centered design and disruptive innovation, working with users to implement technologies that *may change or disrupt the workflow*, in ways expected to improve processes; and finally, is to plan an ICT strategy early and deliberately as a means to avoid regressing towards old ways of working, and instead leveraging and adapting innovative systems to enable transformation towards more integrated models.

## Abbreviations

CCM: Chronic Care Model
CFIR: Consolidated Framework for Implementation Research
DOI: Diffusion of Innovation
eCCM: eHealth Enhanced Chronic Care Model
EHRs: Electronic health records
EMRs: Electronic medical records
FITT: Fit between Individual, Task and Technology
iCOACH: Implementing Integrated Care for Older Adults with Complex Health Needs
ICT: Information communication technology
NPT: Normalization Process Theory
NZ: New Zealand
ON: Ontario
PHRs: Patient personal health records
QC: Quebec
RE-AIM: Reach, Effectiveness, Adoption, Implementation, and Maintenance

## References

[1] Rosella LC, Tiffany F, Walter PW, Andrew C, Heather M, Vivek G (2014). High-cost health care users in Ontario, Canada: demographic, socio-economic, and health status characteristics. BMC Health Serv Res.

[2] Emanuel EJ (2012). Where are the health care cost savings?. JAMA.

[3] Department of Health (2012). Long term conditions compedium of information, third edition.

[4] Heslop L, Athan D, Gardner B, Diers D, Poh BC (2005). An analysis of high-cost users at an Australian public health service organization. Health Serv Manag Res.

[5] Boyd CM, Fortin M (2010). Future of multimorbidity research: how should understanding of multimorbidity inform health system design?. Public Health Rev.

[6] Barnett K, Mercer SW, Norbury M, Watt G, Wyke S, Guthrie B. Epidemiology of multimorbidity and implications for health care, research, and medical education: a cross-sectional study. Lancet. 2012;380(9836):37–43. 10.1016/S0140-6736(12)60240-2 22579043

[7] Marengoni A, Angleman S, Melis R, Mangialasche F, Karp A, Garmen A, Meinow B, Fratiglioni L (2011). Aging with multimorbidity: a systematic review of the literature. Ageing Res Rev.

[8] Bayliss EA, Bosworth HB, Noel PH, Wolff JL, Damush TM, Mciver L (2007). Supporting self-management for patients with complex medical needs: recommendations of a working group. Chronic Illn.

[9] Schaink AK, Kuluski K, Lyons RF, Fortin M, Jadad AR, Upshur R, Wodchis WP (2012). A scoping review and thematic classification of patient complexity: offering a unifying framework. J Comorb.

[10] Courturier Y, Bonin L, Belzile L (2016). Intégration des services en santé Une approche populationnelle.

[11] Kuluski K, Ho JW, Hans PK, Nelson ML (2017). Community care for people with complex care needs: bridging the gap between health and social care. Int J Integr Care.

[12] The Change Foundation (2009). Integrated health care in England: lessons for Ontario.

[13] Protti D (2009). Integrated care needs integration information management and technology. Healthc Q.

[14] Grone O, Garcia-Barbero M (2001). Integrated care: a position paer of the WHO European office for integrated health care services. Int J Integr Care.

[15] Winthereik BR, Bansler JP (2007). Connecting practices: ICT infrastructures to support integrated care. Int J Integr Care.

[16] Dubuc N, Bonin L, Tourigny A, Mathieu L, Couturier Y, Tousignant M, Corbin C, Delli-Colli N, Raîche M (2013). Development of integrated care pathways: toward a care management system to meet the needs of frail and disabled community-dwelling older people. Int J Integr Care.

[17] Barbabella F, Melchiorre MG, Quattrini S, Papa R, Lamura G (2016). How can eHealth improve care for people with multimorbidity in Europe?, in ICARE4EU consortium.

[18] Wagner EH (1998). Chronic disease management: what will it take to improve care for chronic illness?. Eff Clin Pract.

[19] Ouwens M, Wollersheim H, Hermens R, Hulscher M, Grol R (2005). Integrated care programmes for chronically ill patients: a review of systematic reviews. Int J Qual Health Care.

[20] Improving Chronic Illness Care (2003). The chronic care model.

[21] Gee PM, Greenwood DA, Paterniti DA, Ward D, Miller LMS (2015). The eHealth enhanced chronic care model: a theory derivation approach. J Med Internet Res.

[22] Burton LC, Andersen G, Kues IW (2004). Using electronic health records to help coordinate care. Millbank Q.

[23] Institute of Medicine. Key capabilities of an electronic health record system: letter report. Washington DC: The National Academy Press; 2003. 10.17226/10781. 25057672

[24] Pagliari C, Detmer D, Singleton P (2007). Potential of electronic personal health records. BMJ.

[25] McLean S, Sheikh A (2009). Does telehealthcare offer a patient-centred way forward for the community-based management of long-term respiratory disease?. Prim Care Respir J.

[26] Seto E, Leonard KJ, Cafazzo JA, Barnsley J, Masino C, Ross HJ (2012). Mobile phone-based telemonitoring for heart failure management: a randomized controlled trial. J Med Internet Res.

[27] Townsend A, Adam P, Li LC, McDonald M, Backman CL (2013). Exploring eHealth ethics and multi-morbidity: protocol for an interview and focus group study of patient and health care provider views and experiences of using digital media for health purposes. JMIR Res Protoc.

[28] Rogers EM (2003). Diffusion of innovations.

[29] May C, Mair F, Finch T, MacFarlane A, Dowrick C, Treweek S (2009). Development of a theory of implementation and integration: normalization process theory. Implement Sci.

[30] Glasgow RE, McKay HG, Piette JD, Reynolds KD (2001). The RE-AIM framework for evaluating interventions: what can it tell us about approaches to chronic illness management?. Patient Educ Couns.

[31] Ammenwerth E, Iller C, Mahler C (2006). IT-adoption and the ineraction of task, technology and individuals: a fit framework and a case study. BMC Med Inform Decis Mak.

[32] Damschroder LJ, Aron DC, Keith RE, Kirsh SR, Alexander JA, Lowery JC (2009). Fostering implementation of health services research findings into practice: a consolidated framework for advancing implementation science. Implement Sci.

[33] Zhang X, Yu P, Yan J, Spil ITA (2015). Using diffusion of innovation theory to understand the factors impacting patient acceptance and use of consumer e-health innovations: a case study in a primary care clinic. BMC Health Serv Res.

[34] Bouamrane M-M, Mair FS (2014). Using diffusion of innovation theory to understand the factors impacting patient acceptance and use of consumer e-health innovations: a case study in a primary care clinic. BMC Med Inform Decis Mak.

[35] Glasgow RE, Dickinson P, Fisher L, Christiansen S, Toobert DJ, Bender BG, Dickinson LM, Jortberg B, Estabrooks PA (2011). Use of RE-AIM to develop a multi-media facilitation tool for the patient-centered medical home. Implement Sci.

[36] Steele Gray C, Gill A, Khan AI, Hans PK, Kuluski K, Cott C. The electronic patient reported outcome tool: testing usability and feasibility of a mobile app and portal to support care for patients with complex chronic disease and disability in primary care settings. JMIR mHealth uHealth. 2016;4(2):e58. 10.2196/mhealth.5331 PMC4911509 27256035

[37] Varsi C, Ekstedt M, Gammon D, Ruland CM (2015). Using the consolidated framework for implementation research to identify barriers and facilitators for the implementation of an internet-based patient-provider communication service in five settings: a qualitative study. JMIR.

[38] Steele Gray C, Wodchis WP, Baker GR, Carswell P, Kenealy T, McKillop A (2018). Mapping for conceptual clarity: exploring implementation of integrated community-based primary health care from a whole systems perspective. Int J Integr Care.

[39] Cresswell JW. Research design: qualitative, quantitative, and mixed methods approaches. 2nd ed. Thousand Oaks: SAGE Publications; 2003.

[40] Yin RK (2009). Case study research, design and method.

[41] International Journal of Integrated Care (2017). Special Collection iCOACH. Implementing Integratec Care for Older Adults with Complex Health Needs.

[42] Kuluski K, Sheridan N, Kenealy T, Breton M, McKillop A, Shaw J, Nie JX, Upshur RE, Baker GR, Wodchis WP (2017). “On the margins and not the mainstream:” case selection for the implementation of community based primary health care in Canada and New Zealand. Int J Integr Care.

[43] Dolovich L, Oliver D, Lamarche L, Agarwal G, Carr T, Chan D, et al. A protocol for a pragmatic randomized controlled trial using the health teams advancing patient experience: strengthening quality (Health TAPESTRY) platform approach to promote person-centred primary health care for older adults. Impelment Sci. 2016;11:49. 10.1186/s13012-016-0407-5 PMC4820854 27044360

[44] Breton M, Steele Gray C, Sherian N, Shaw J, Parsons J, Wankah P, et al. Implementing community based primary healthcare for older adults with complex needs in Quebec, Ontario and New-Zealand: describing nine cases. Int J Integr Care. 2017;17(2):12. 10.5334/ijic.2506 PMC5624082 28970753

[45] Tenbensel T, Miller F, Breton M, Couturier Y, Morton-Chang F, Ashton T (2017). How do policy and institutional settings shape opportunities for community-based primary health care? A comparison of Ontario, Québec and New Zealand. Int J Integr Care.

[46] Clark E (2016). Value and opportunities created by Ontario’s digital health asset.

[47] Beardwood JP, Kerr JA (2003). Coming soon to a health sector near you: an advance look at the new Ontario Personal Health Information Protection Act (PHIPA). Healthc Q.

[48] Schoen C, Osborn R, Squires D, Doty M, Rasmussen P, Pierson R, Applebaum S (2012). A survey of primary care doctors in ten coutnries shows progress in use of health information technology, less in other areas. Health Aff.

[49] Evans J, Grudniewicz A, Baker GR, Wodchis W (2016). Organizational context and capabilities for integrating care: a framework for improvement. Int J Integr Care.

[50] Sandelowski M (2000). Whatever happened to qualitative description?. Res Nurs Health.

[51] Green J, Thorogood N, Silverman D (2014). Chapter 8: Beginning data analysis. Qualitative methods for health research.

[52] Sandelowski M (1986). The problem of rigor in qualitative research. Adv Nurs Sci.

[53] Christensen CM (1997). The innovators dilemma: when new technologies cause great firms to fail.

[54] Christensen CM, Bohmer R, Kenagy J (2000). Will disruptive innovations cure health care?. Harv Bus Rev.

[55] Kirch DG (2010). The healthcare innovation zone: a platform for true reform. JAMA.

[56] Brook RH (2009). Disruption and innovation in health care. JAMA.

[57] Fernández-Alemán JL, Señor IC, Lozoya PÁO, Toval A (2013). Security and privacy in electronic health records: a systematic literature review. J Biomed Inform.

[58] Goldfarb A, Tucker C (2012). Privacy and innovation. Innov Policy Econ.

[59] Hassol A, Walker JM, Kidder D, Rokita K, Young D, Pierdon S (2004). Patient experiences and attitudes about access to a patient electroinc health record and linked web messaging. J Am Med Inform Assoc.

[60] Steele Gray C, Miller D, Kuluski K, Cott C. Tying eHealth tools to patient needs: exploring the use of eHealth for community-dwelling patients with complex chronic disease and disability. JMIR Res Protoc. 2014;3(4):e67. 10.2196/resprot.3500 PMC4260075 25428028

[61] Byrne CM, Mercincavage LM, Bouhaddou O, Bennett JR, Pan EC, Botts NE (2014). The Department of Veterans Affairs’ (VA) implementation of the virtual lifetime electronic record (VLER): findings and lessons learned from health information exchange at 12 sites. Int J Med Inform.

[62] Villalba E, Casas I, Abadie F, Lluch M (2013). Integrated personal health and care services deployment: experiences in eight European countries. Int J Med Inform.

[63] Hoerbst A, Schweitzer M (2015). A systematic investigation on barriers and critical success factors for clinical information systems in integrated care settings. Yearb Med Inform.

[64] Steele Gray C, Mercer S, Palen T, McKinstry B, Hendry A. eHealth advances in support of people with complex care needs: case examples from Canada, Scotland and the US. Healthc Q. 2016;19(2):29–37. 10.12927/hcq.2016.24696 27700971

[65] Townsend M (2014). Learning from Kaiser Permanente: integrated systems and healthcare improvement in Canada.

[66] Subramanyam R, Weisstein FL, Krishnan MS (2010). User participation in software development projects. Commun ACM.

[67] Devi KR, Sen AM, Hemachandran K (2012). A working framework for the user-centered design approach and a survey of the available methods. Int J Sci Res Publ.

[68] Chan J, Shojania KG, Easty AC, Etchells EE (2011). Does user-centred design affect the efficiency, usability and safety of CPOE order sets?. J Am Med Inform Assoc.

[69] Kujala S (2003). User involvement: a review of the benefits and challenges. Behav Inf Technol.

[70] Sultan N (2015). Reflective thoughts on the potential and challenges of wearable technology for healthcare provision and medical education. Int J Inf Manag.

[71] Nasi G, Cucciniello M, Guerrazzi C (2015). The role of mobile technologies in health care processes: the case of cancer supportive care. JMIR.

[72] Goodwin N (2010). The state of telehealth and telecare in the UK: prospects for integrated care. J Integr Care.

[73] Schwamm L (2014). Telehealth: seven strategies to successfully implement disruptive technology and transform health care. Health Aff (Millwood).

[74] Greve HR, Taylor A (2000). Innovations as catalysts for organizational change: shifts in organizational cognition and search. Adm Sci Q.

[75] Ziestma C, Lawrence TB (2010). Institutional work in the transformation of an organizational field: the interplay of boundary work and practice work. Adm Sci Q.

[76] Leutz W (1999). Five laws for integrating medical and social services: lessons from the United States and the United Kingdom. Milbank Q.

[77] Contel JC, Ledesma A, Blay C, Mestre AG, Cabezas C, Puigdollers M (2015). Chronic and integrated care in Catalonia. Int J Integr Care.

[78] Generalitat de Catalunya Departament de Salut, ICT strategy in the Catalan Healthcare and Socialcare system, I. Services, Editor. Generalitat de Catalunya Departament de Salut: Catalonia; 2017.

[79] Michie S, Johnston M, Abraham C, Lawton R, Parker D, Walker A, on behalf of the ‘Psychological Theory’ Group (2005). Making psychological theory useful for implementing evidence based practice: a consensus approach. Qual Saf Health Care.

